# METTL3 aggravates cell damage induced by *Streptococcus pneumoniae* via the NEAT1/CTCF/MUC19 axis

**DOI:** 10.1002/kjm2.12843

**Published:** 2024-05-16

**Authors:** Dong‐Bo Ma, Hui Zhang, Xi‐Ling Wang, Qiu‐Ge Wu

**Affiliations:** ^1^ Department of Respiratory and Critical Care Medicine The First Affiliated Hospital of Zhengzhou University Zhengzhou City China

**Keywords:** alveolar epithelial cells, LncRNA NEAT1, METTL3, MUC19, *Streptococcus pneumoniae*

## Abstract

Disruption of the alveolar barrier can trigger acute lung injury. This study elucidated the association of methyltransferase‐like 3 (METTL3) with *Streptococcus pneumoniae* (SP)‐induced apoptosis and inflammatory injury of alveolar epithelial cells (AECs). AECs were cultured and then infected with SP. Furthermore, the expression of METTL3, interleukin (IL)‐10, IL‐6, tumor necrosis factor‐alpha (TNF‐α), monocyte chemoattractant protein‐1 (MCP‐1), long noncoding RNA nuclear paraspeckle assembly transcript 1 (NEAT1), mucin 19 (MUC19), N6‐methyladenosine (m6A), and NEAT1 after m6A modification were detected by qRT‐PCR, Western blot, and enzyme‐linked immunosorbent, m6A quantification, and methylated RNA immunoprecipitation‐qPCR analyses, respectively. Moreover, the subcellular localization of NEAT1 was analyzed by nuclear/cytosol fractionation assay, and the binding between NEAT1 and CCCTC‐binding factor (CTCF) was also analyzed. The results of this investigation revealed that SP‐induced apoptosis and inflammatory injury in AECs and upregulated METTL3 expression. In addition, the downregulation of METTL3 alleviated apoptosis and inflammatory injury in AECs. METTL3‐mediated m6A modification increased NEAT1 and promoted its binding with CTCF to facilitate MUC19 transcription. NEAT1 or MUC19 overexpression disrupted their protective role of silencing METTL3 in AECs, thereby increasing apoptosis and inflammatory injury. In conclusion, this is the first study to suggest that METTL3 aggravates SP‐induced cell damage via the NEAT1/CTCF/MUC19 axis.

## 1 INTRODUCTION

In the lungs, the epithelium comprises alveolar epithelial cells (AECs), a key component of lung homeostasis. AECs provide a wide surface area for gas exchange, sequestering inhaled foreign substances, and regulating the transport of water and ions.^1^ Furthermore, the alveolar epithelium provides a protective mechanical barrier against inhaled pathogens that can cause pneumonia, and the disruption of the alveolar epithelial barrier by proinflammatory factors and bacteria may trigger bacterial pneumonia and acute lung injury (ALI).^2^ *Streptococcus pneumoniae* (SP) is a Gram‐positive bacterium that can spread from the nasopharynx to the lungs and induce bacterial pneumonia, a common cause of serious infections worldwide.^3^, ^4^ Patients with pneumococcal infection have the following manifestations intestinal chills, fever, malaise, cough, and difficulty breathing and may progress to acute respiratory failure, septic shock, multiorgan failure, and death.^5^ Currently, antibiotics are used to treat bacterial pneumonia; however, because of the increasing antibiotic resistance, treatment of different bacterial infections has become significantly challenging.^6^ In this study, the molecular mechanism that regulates the inflammation of AECs was determined using a cell model of SP‐induced pneumonia to find new targets for its treatment.

N6‐methyladenosine (m6A) is the most common internal posttranscriptional modification on message RNAs (mRNAs) and regulates pre‐mRNA splicing, mRNA translation, stability, structure, export, and decay.^7^ It has been identified that m6A methylation is catalyzed by methyltransferase‐like 3 (METTL3), which acts as a writer of m6A modification and also targets methylated mRNA and promotes mRNA translation.^8^ The literature has indicated markedly increased METTL3 expression in pediatric pneumonia patients and cell models.^9^ Furthermore, METTL3 downregulation can alleviate lipopolysaccharide (LPS)‐induced microglial inflammation and improve neonatal pneumonia by affecting genes mRNA m6A levels.^10^ However, the effect and mechanism of METTL3 in SP‐induced AECs remains undetermined.

Nuclear enriched abundant transcript 1 (NEAT1) is a long noncoding (lnc) RNA that is located on chromosome 11. Moreover, it is one of the most abundant lncRNAs in the nuclear paraspeckles of mammalian cells and can target multiple genomic regions of various cells.^11^ It has been reported that NEAT1 has multiple m6A modification sites, and METTL3‐mediated m6A modification induces the overexpression of NEAT1.^12^ In addition, in the LPS‐induced lung inflammatory cell model, NEAT1 expression was significantly increased by LPS in a concentration‐dependent manner, which decreased cell viability and induced cell apoptosis.^13^ The subcellular localization prediction of NEAT1 revealed that it is primarily localized in the nucleus, where it can bind to transcription factors and promote the transcription of downstream genes.^14^ Therefore, this research also investigated the transcription factors and downstream mechanisms of NEAT1.

Mucins (MUCs) are glycoproteins coated with thick glycans and their inner epithelial surface functions to remove microorganisms and establish protective barriers.^15^ Furthermore, MUC5AC and MUC5B are the primary solid components of the mucus layer, which participate in airway defense. Moreover, abnormal MUC expression is closely related to inflammation and lung diseases.^16^ In the lungs, there are four secreted MUCs: MUC2, MUC5AC, MUC5B, and MUC19.^17^ It has been indicated that in the lungs of human metapneumovirus (HMPV)‐infected mice, MUC19 is significantly increased and its knockdown reduces HMPV‐induced lung inflammation.^18^ Currently, the research on the specific MUC19 pathways in pneumonia is limited.

This investigation elucidated the effect of METTL3 on SP‐induced AECs and explored the downstream pathways of METTL3 to provide a new theoretical basis for the treatment of bacterial pneumonia.

## 2 MATERIALS AND METHODS

### 2.1 Cell culture and grouping

Human pulmonary alveolar epithelial cells (HPAEpiC) were purchased from Shanghai Huiying Biological Company (Shanghai, China) and cultured in RPMI 1640 medium augmented with 10% fetal bovine serum at 37°C with 5% CO_2_. *Streptococcus pneumoniae* (SP; 49619; ATCC, Manassas, VA, USA) was provided by Shanghai Xinshuo Biotechnology Co., Ltd (Shanghai, China) and cultured on tryptic soy agar with 0.5% defibrinated sheep blood (Qingdao Haibo Biological Co., Ltd., China). Before the infection, for 12 h, SP was treated with Todd‐Hewitt broth with 0.5% yeast (Qingdao Haibo Biological Co., Ltd.). Log‐phase HPAEpiC cells were divided into Ctrl, SP, SP + si‐NC, SP + si‐METTL3, SP + si‐METTL3 + NC, SP + si‐METTL3 + NEAT1, and SP + si‐METTL3 + MUC19 groups. In gene intervention groups, HPAEpiC cells were transfected with METTL3 small interfering RNA (siRNA), pcDNA‐NEAT1, pcDNA‐MUC19, CCCTC‐binding factor (CTCF) siRNA, or corresponding controls (Shanghai GenePharma Co., Ltd., China). After transfection for 48 h using Lipofectamine™ 2000 reagent (ThermoFisher, Waltham, MA, USA), the six groups were infected with SP at 1 × 10^8^ colony‐forming unit (CFU)/mL for 24 h. The Ctrl group consisted of untreated HPAEpiC cells, while the SP group comprised HPAEpiC cells treated with SP for 24 h without transfection.

### 2.2 Flow cytometry

Cell staining was performed using the TransDetect® Annexin V‐fluorescein isothiocyanate (FITC)/propidium iodide (PI) Cell Apoptosis Detection Kit (TransGen Biotech Co., Ltd, Beijing, China). Briefly, 1 × 10^6^ cells were collected, resuspended in an ice‐cold phosphate‐buffered saline trice, mixed with 500 μL of binding buffer, and then were sequentially treated with 10 μL of PI and 5 μL of Annexin V‐FITC for 15 min in the dark at room temperature. Flow cytometric analysis was conducted using the NovoCyte Advanteon B4 flow cytometer and NovoSampler Q software (Agilent Technologies Co., Ltd, Beijing, China).

### 2.3 Enzyme‐linked immunosorbent assay (ELISA)

After grouping and respective treatments, the supernatant of cells from each group was collected and used to detect the levels of tumor necrosis factor (TNF)‐α (ab181421, Abcam, Cambridge, MA, USA), interleukin (IL)‐6 (ab178013, Abcam), monocyte chemoattractant protein (MCP)‐1 (ab179886, Abcam), and IL‐10 (ab185986, Abcam). The protocol provided by the respective assay kits was followed.

### 2.4 Cell counting kit (CCK)‐8 assay

Cells were resuspended (1 × 10^5^ cells/mL) were seeded in 96‐well plates at a concentration of 100 μL/well. After 48 h, cell viability was assessed by adding 10 μL of CCK‐8 reagent (AmyJet Technology Co., Ltd., Wuhan, China) in each well for 2 h at 37°C.

### 2.5 m6A quantification

Total RNA was isolated using TRIzol (Invitrogen, Carlsbad, CA, USA), per the kit's protocol. Then the relative abundance of m6A in RNA was assessed via the EpiQuik m6A RNA methylation quantification kit (Colorimetric) (ab185912, Abcam), per the kit's protocol. Briefly, RNA (200 ng) was incubated with an anti‐m6A antibody‐containing solution. The m6A levels were quantified using colorimetric assay at 450 nm absorbance.

### 2.6 Methylated RNA immunoprecipitation‐quantitative polymerase chain reaction (MeRIP‐qPCR)

For MeRIP‐qPCR, the Magna MeRIP™ m6A kit (Millipore, Billerica, Massachusetts, USA) was employed, and the method provided by the manufacturer was followed. Briefly, the extracted total RNA was mixed with the MeRIP reagent. The Magna ChIP protein A/G magnetic beads were resuspended and treated overnight with anti‐m6A (ab208577, Abcam) or anti‐immunoglobulin G (IgG) (ab172730, Abcam) antibodies at 4°C. Subsequently, the beads were eluted from the complexes, and RNA levels were quantified using RT‐qPCR.

### 2.7 RNA stability assay

Cells were seeded in 6‐well plates and were treated with Actinomycin D (5 μg/mL, Sigma‐Aldrich, St. Louis, MO, USA). Then at specified time points (0, 4, 8, and 12 h), cells were collected and their total RNA was extracted for RT‐qPCR analysis.

### 2.8 Nuclear‐cytoplasmic fractionation assay

The cell localization analysis was performed via the PARIS Kit (Invitrogen), per the manufacturer's guide. Briefly, cell nuclei and cytoplasm were separated and quantified via RT‐qPCR. U6 and GAPDH were used as positive controls for nuclear and cytoplasm, respectively.

### 2.9 RIP assay

Total cellular RNA was isolated using TRIzol. The anti‐CTCF (ab300639, Abcam) and anti‐IgG (ab172730, Abcam) antibodies were coupled to protein A/G magnetic beads in the IP buffer (140 mM NaCl, 1% NP‐40, 2 mM Ethylene Diamine Tetraacetic Acid, 20 mM Tris pH 7.5) and incubated overnight at 4°C. Then, the acquired RNA was treated with the antibodies in the IP buffer before RNA precipitate elution from the beads. Subsequently, precipitated RNA and input total RNA were eluted to calculate the relative enrichment.

### 2.10 RNA pull‐down assay

For this assay, the MagCapture™ RNA pull‐down assay kit (Whatman Co., Ltd., Maidstone, Kent, UK) was used, and the method provided in the kit was followed. Briefly, after lysis, the cells were incubated with biotinylated NEAT1 and control probes. Streptavidin‐coated magnetic beads were resuspended and incubated with the probes overnight at 4°C. Lastly, these beads were eluted from the RNA‐protein complexes and analyzed.

### 2.11 RT‐qPCR

Total cellular RNA was extracted using TRIzol Reagent (Invitrogen) and then used to prepare complementary DNA (cDNA), according to the instructions of the PrimeScript™ RT reagent Kit (Takara, Dalian, China). Then, RT‐qPCR was performed by following the SYBR Green qPCR kit (ThermoFisher) protocol. GAPDH was used as an internal control. The relative fold expression of the target gene was calculated using the 2^−ΔΔCt^ method^19^ and normalized to the control. Table 1 enlists the primer sequences used in this experiment.

**TABLE 1:** PCR primer sequences.

Gene	Sequence (5′‐3′)
METTL3	F: TGGGGGTATGAACGGGTAGA
	R: TGGTTGAAGCCTTGGGGATT
LncRNA NEAT1	F: CCACAACGCAGATTGATGCC
	R: GAAACGCACAAGAAGGCAGG
MUC19	F: GTTGACCCCAGTGCTTACCA
	R: CTACACGCCTCTGCGTACAT
IL‐10	F: TTGCCTGGTCCTCCTGACTG
	R: GAAGCATGTTAGGCAGGTTGC
IL‐6	F: TTCGGTCCAGTTGCCTTCTC
	R: TCTTCTCCTGGGGGTACTGG
TNF‐α	F: GCTGCACTTTGGAGTGATCG
	R: TCACTCGGGGTTCGAGAAGA
MCP‐1	F: GAAAGTCTCTGCCGCCCTT
	R: GGTGACTGGGGCATTGATTG
CTCF	F: AACCAGCCCAAACAGAACCA
	R: TCCTCTTCCTCTCCCTCTGC
GAPDH	F: GTCAAGGCTGAGAACGGGAA
	R: TCGCCCCACTTGATTTTGGA

### 2.12 Western blot assay

The proteins were extracted by lysing cells with lysis buffer (Beyotime, Shanghai, China), quantified via the bicinchoninic acid protein assay kit (Beyotime), separated by SDS‐PAGE gel electrophoresis, and then transferred onto polyvinylidene fluoride membranes. The membranes were then blocked with 5% nonfat milk, treated overnight with primary METTL3 (1:1000, ab195352, Abcam), Bax (1:1000, ab32503, Abcam), Bcl‐2 (1:2000, ab182858, Abcam), CTCF (1:1000, ab300639, Abcam), and GAPDH (1:2500, ab9485, Abcam) antibodies at 4°C and then tagged with secondary antibody (1:2000, ab205718, Abcam). The protein bands were visualized using the enhanced chemiluminescence method (Millipore). GAPDH was used as an internal control. The intensity of the bands was semi‐quantified using ImageJ software (version 1.52r; National Institutes of Health).

### 2.13 Statistical methods

All data were analyzed and plotted using SPSS 21.0 (IBM SPSS Statistics, Armonk, NY, USA) and GraphPad Prism 8.0 (GraphPad Software Inc., San Diego, CA, USA) software. First, it was identified if the data were normally distributed or homogeneous by the normality and homogeneity of variance tests. The *t*‐test was used for comparisons between two groups, while one‐way or two‐way analysis of variance (ANOVA) analysis was used for comparisons among multiple groups, followed by Tukey's multiple comparisons test post hoc analysis. The p‐value was generated using the two‐tailed test, and *p* < 0.05 was deemed statistically significant, while *p* < 0.01 was considered highly statistically significant.

## 3 RESULTS

### 3.1 SP induces apoptosis and inflammatory injury in AECs and promotes METTL3 expression

**FIGURE 1:**
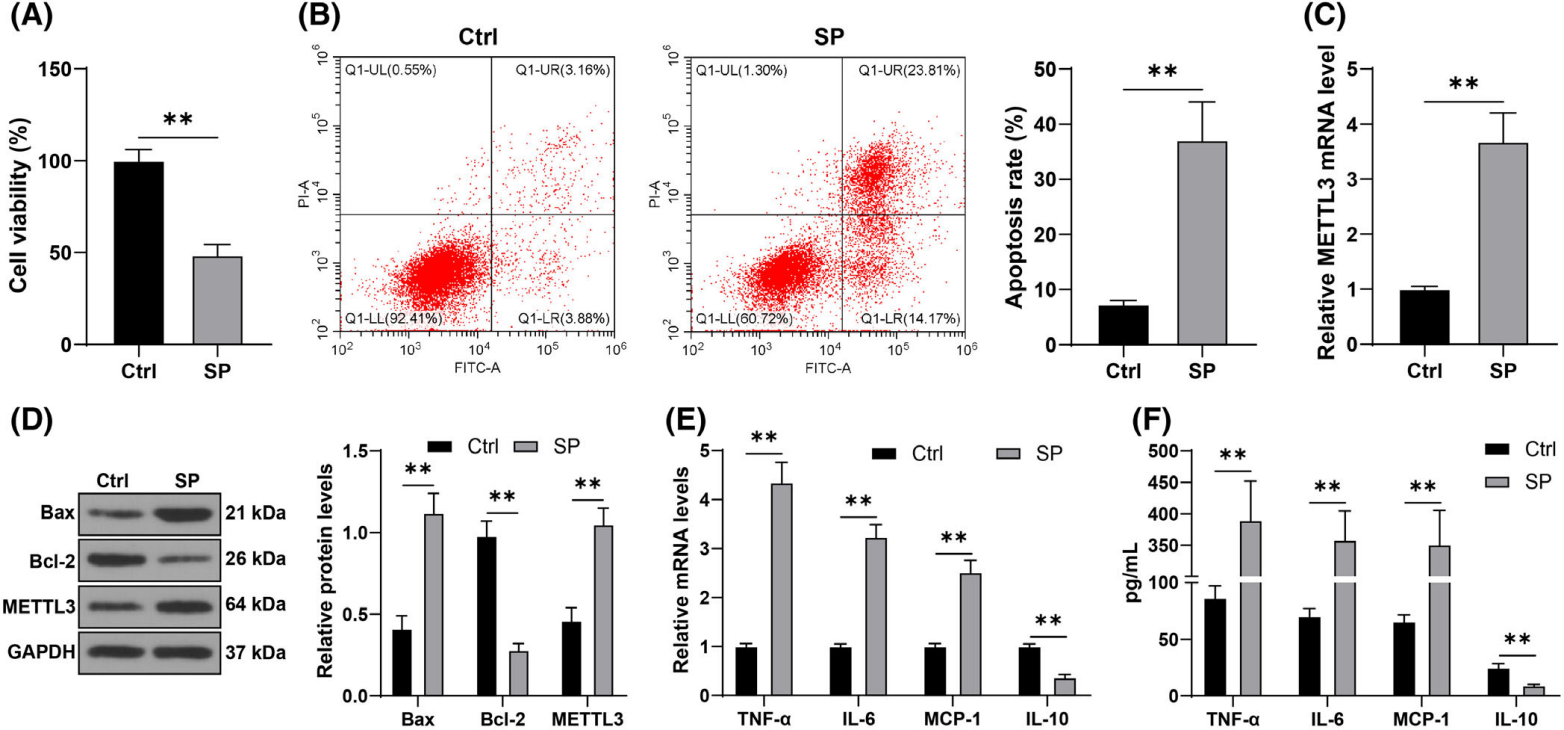
SP induces apoptosis and inflammatory injury in AECs and promotes METTL3 expression. AECs were infected with SP, and the untreated cells were used as the control (Ctrl). (A): Cell viability was detected using CCK‐8 assay. (B): The apoptosis rate was measured by flow cytometry. (C): mRNA levels of METTL3 were measured by RT‐qPCR. (D): Western blot analysis detected the cellular levels of Bax, Bcl‐2, and METTL3 in proteins. (E, F): Levels of TNF‐α, IL‐6, MCP‐1, and IL‐10 were measured by RT‐qPCR and ELISA. Each experiment was repeated thrice and the data were presented as mean ± SD. Data comparisons between two groups in panels A‐C were analyzed using a *t*‐test, while multiple group comparisons in panels D‐F were analyzed using two‐way ANOVA, followed by Tukey's multiple comparisons test. ** *p* < 0.01.

The role and mechanism of METTL3 in SP‐induced AECs remain unclear. In this study, cells were infected with SP, and it was revealed that the cell viability in the SP group was significantly decreased (*p* < 0.05, Figure 1A), while the apoptosis rate was significantly increased (*p* < 0.05, Figure 1B) compared to the Ctrl group. Furthermore, after SP induction, the level of the apoptosis‐related Bax protein was increased, while that of Bcl‐2 was decreased (*p* < 0.05, Figure 1D). Additionally, the levels of TNF‐α, IL‐6, and MCP‐1 were increased after SP infection, while the level of IL‐10 was decreased (*p* < 0.05, Figure 1E,F), suggesting that SP‐induced apoptosis and inflammatory injury. In addition, SP also significantly upregulated METTL3 expression (*p* < 0.05, Figure 1C,D).

### 3.2 METTL3 downregulation alleviates cell damage induced by SP

**FIGURE 2:**
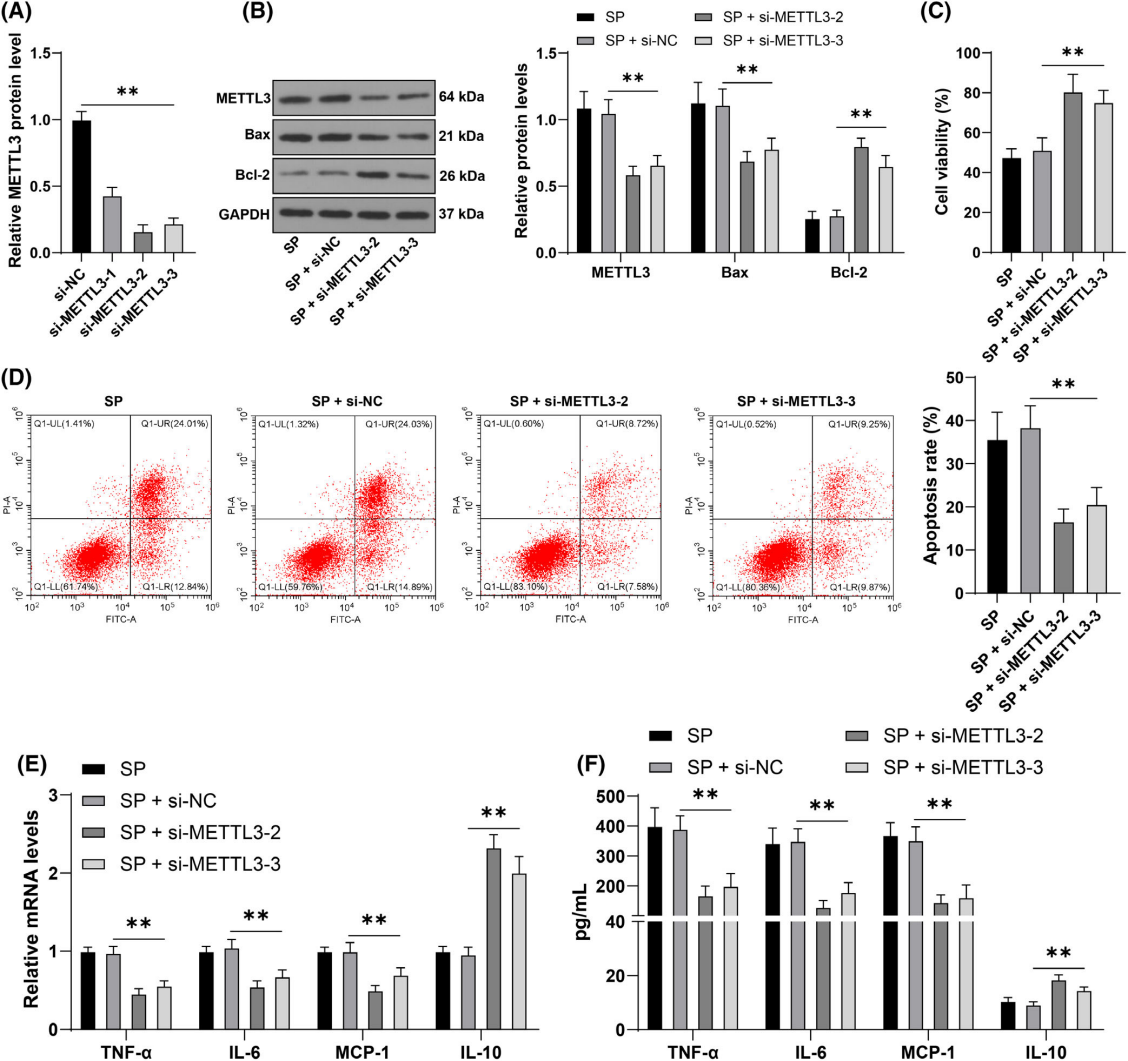
Downregulation of METTL3 alleviates cell damage induced by SP. Cells were transfected with si‐METTL3, and negative control cells were transfection with si‐NC. AECs were infected with SP. (A): mRNA levels of METTL3 were measured by RT‐qPCR. (B): Western blot analysis detected the cellular levels of Bax, Bcl‐2, and METTL3 in proteins. (C): Cell viability was detected using CCK‐8 assay. (D): Apoptosis rate was measured by flow cytometry. (E, F): Levels of TNF‐α, IL‐6, MCP‐1, and IL‐10 were measured by RT‐qPCR and ELISA. Each experiment was repeated thrice, and the data were presented as mean ± standard deviation. Data comparisons among multiple groups in panels A, C, and D were analyzed using one‐way ANOVA, while comparisons in panels B, E, and F were analyzed using two‐way ANOVA, followed by Tukey's multiple comparisons test. ** *p* < 0.01.

For further validation, the expression of METTL3 was downregulated by transfecting METTL3 siRNA (*p* < 0.05, Figure 2A,B) and si‐METTL3‐2 and si‐METTL3‐3 was selected with better intervention efficiency. The results indicated that with decreasing METTL3 expression, the cell viability increased (*p* < 0.05, Figure 2C) and the apoptosis rate decreased (*p* < 0.05, Figure 2D). Furthermore, after METTL3 downregulation, the levels of Bax were decreased, while that of Bcl‐2 was increased (*p* < 0.05, Figure 2B). Moreover, si‐METTL3 transfection decreased levels of TNF‐α, IL‐6, and MCP‐1, whereas increased IL‐10 levels compared to si‐NC transfection (*p* < 0.05, Figure 2E,F). In summary, METTL3 downregulation alleviates apoptosis and inflammatory damage in AECs induced by SP.

### 3.3 METTL3‐mediated m6A modification increases NEAT1 expression

**FIGURE 3:**
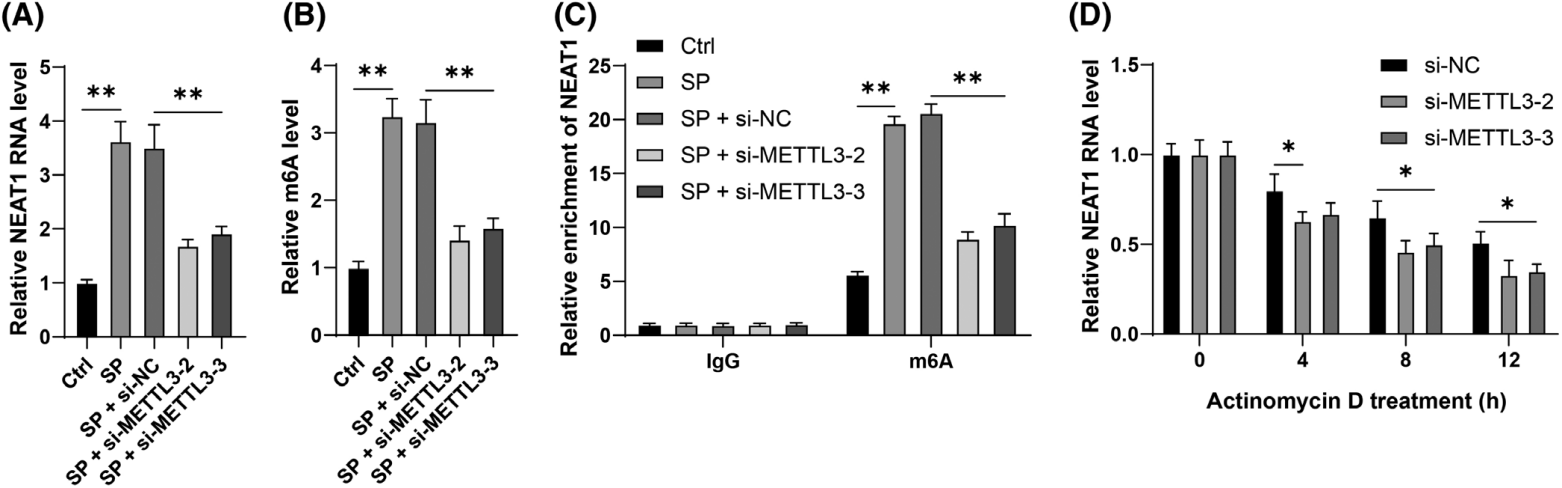
METTL3‐mediated m6A modification increases lncRNA NEAT1 expression. (A): RT‐qPCR was performed to assess the levels of lncRNA NEAT1. (B): Analysis of m6A levels in cells. (C): MeRIP‐qPCR analysis of m6A modification on lncRNA NEAT1 RNA levels. (D): RNA stability of lncRNA NEAT1 after actinomycin D treatment was examined by RT‐qPCR. Each experiment was repeated thrice and the data were presented as mean ± standard deviation. Data comparisons among multiple groups in panels A and B were analyzed using one‐way ANOVA, while comparisons in panels C and D were analyzed using two‐way ANOVA, followed by Tukey's multiple comparisons test. * *p* < 0.05, ** *p* < 0.01.

The literature has indicated that METTL3‐mediated m6A modification induces abnormal expression of the lncRNA NEAT1.^12^ Furthermore, NEAT1 has been observed to be highly expressed in pneumonia,^13^ consistent with this study (*p* < 0.01, Figure 3A). It was hypothesized that NEAT1 is a downstream mechanism of METTL3. m6A quantitative analysis revaled that m6A was reduced in METTL3‐downregulated cells (*p* < 0.01, Figure 3B). Further analysis revealed m6A upregulation in NEAT1 RNA, which was subsequently downregulated after METTL3 knockdown (*p* < 0.01, Figure 3C). Moreover, RNA stability analysis indicated that METTL3 downregulation decreased the NEAT1 stability (*p* < 0.01, Figure 3D) and NEAT1 expression (*p* < 0.01, Figure 3A). In conclusion, these findings suggest that METTL3‐mediated m6A modification may increase NEAT1 expression.

### 3.4 NEAT1 overexpression attenuates the protective effect of METTL3 downregulation on cell damage

**FIGURE 4:**
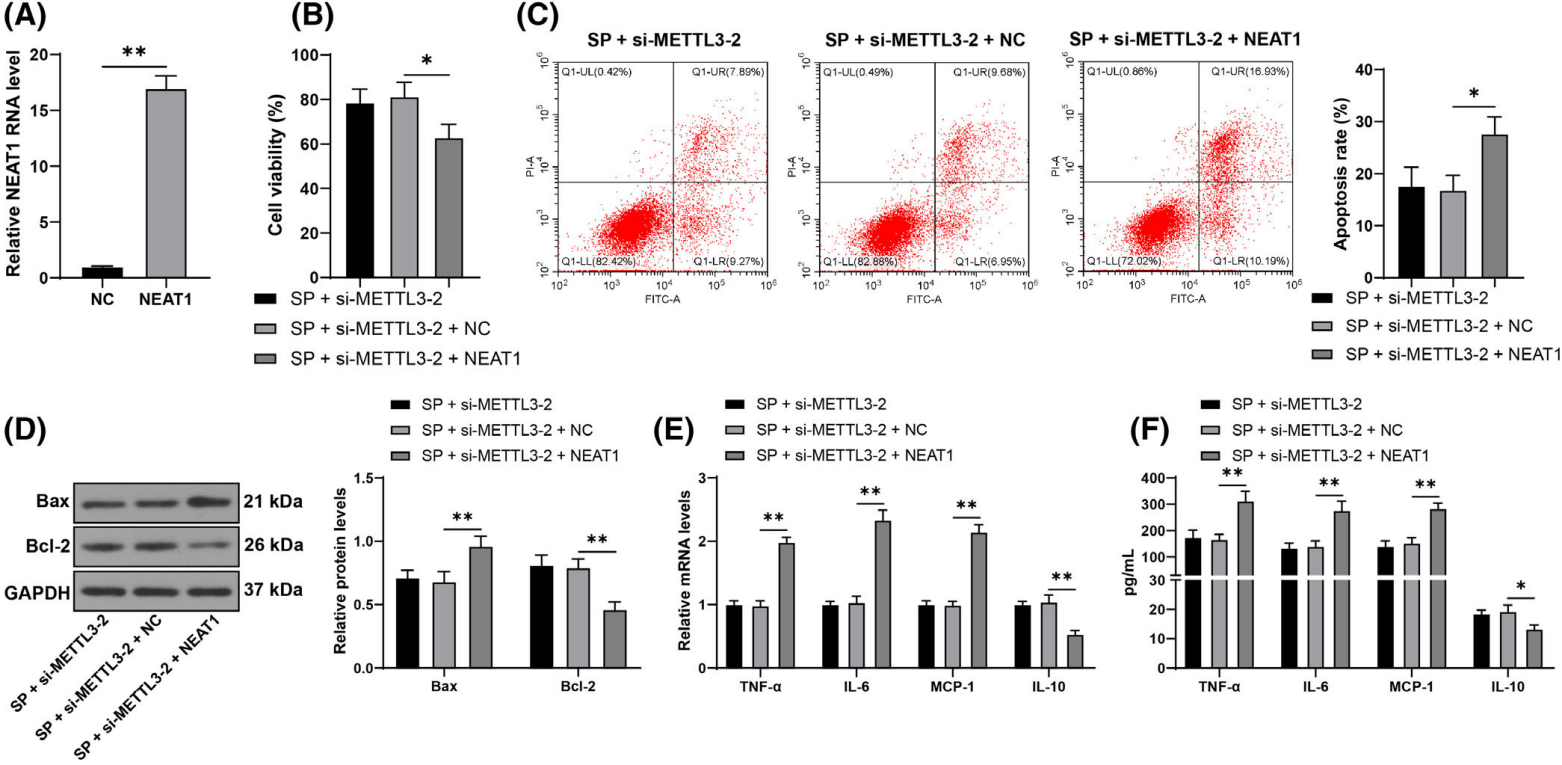
NEAT1 overexpression alleviated the protective effect of METTL3 downregulation on cell damage. Cells were transfected with pcDNA‐NEAT1 (NEAT1), and the negative control was transfected with pcDNA‐NC (NC). AECs were infected with SP. (A): LncRNA NEAT1 levels were measured by RT‐qPCR. (B): Cell viability was detected using CCK‐8 assay. (C): Apoptosis rate was measured by flow cytometry. (D): Levels of Bax and Bcl‐2 in cells were assessed by Western blot assay. (E, F): Levels of TNF‐α, IL‐6, MCP‐1, and IL‐10 were measured by RT‐qPCR and ELISA. Each experiment was repeated thrice, and the data were presented as mean ± standard deviation. Data comparisons between the two groups in panel A were analyzed using a *t*‐test, while comparisons among multiple groups in panels B and C were analyzed using one‐way ANOVA, while multiple group comparisons in panels D‐F were analyzed using two‐way ANOVA, followed by Tukey's multiple comparisons test. * *p* < 0.05, ** *p* < 0.01.

The functional rescue experiments were performed by transfecting pcDNA‐NEAT1 (NEAT1) to upregulate NEAT1 expression (*p* < 0.05, Figure 4A), in combination with si‐METTL3‐2. It was found that NEAT1 upregulation decreased cell viability and increased apoptosis in SP‐induced cells (*p* < 0.05, Figure 4B–D). Compared to METTL3 downregulation alone, the combined experimental group showed increased levels of TNF‐α, IL‐6, and MCP‐1 in cells and decreased levels of IL‐10 (*p* < 0.05, Figure 4E,F). In summary, NEAT1 overexpression attenuates the protective effect of METTL3 downregulation on cell damage induced by SP.

### 3.5 NEAT1 binds to CTCF and promotes MUC19 transcription

**FIGURE 5:**
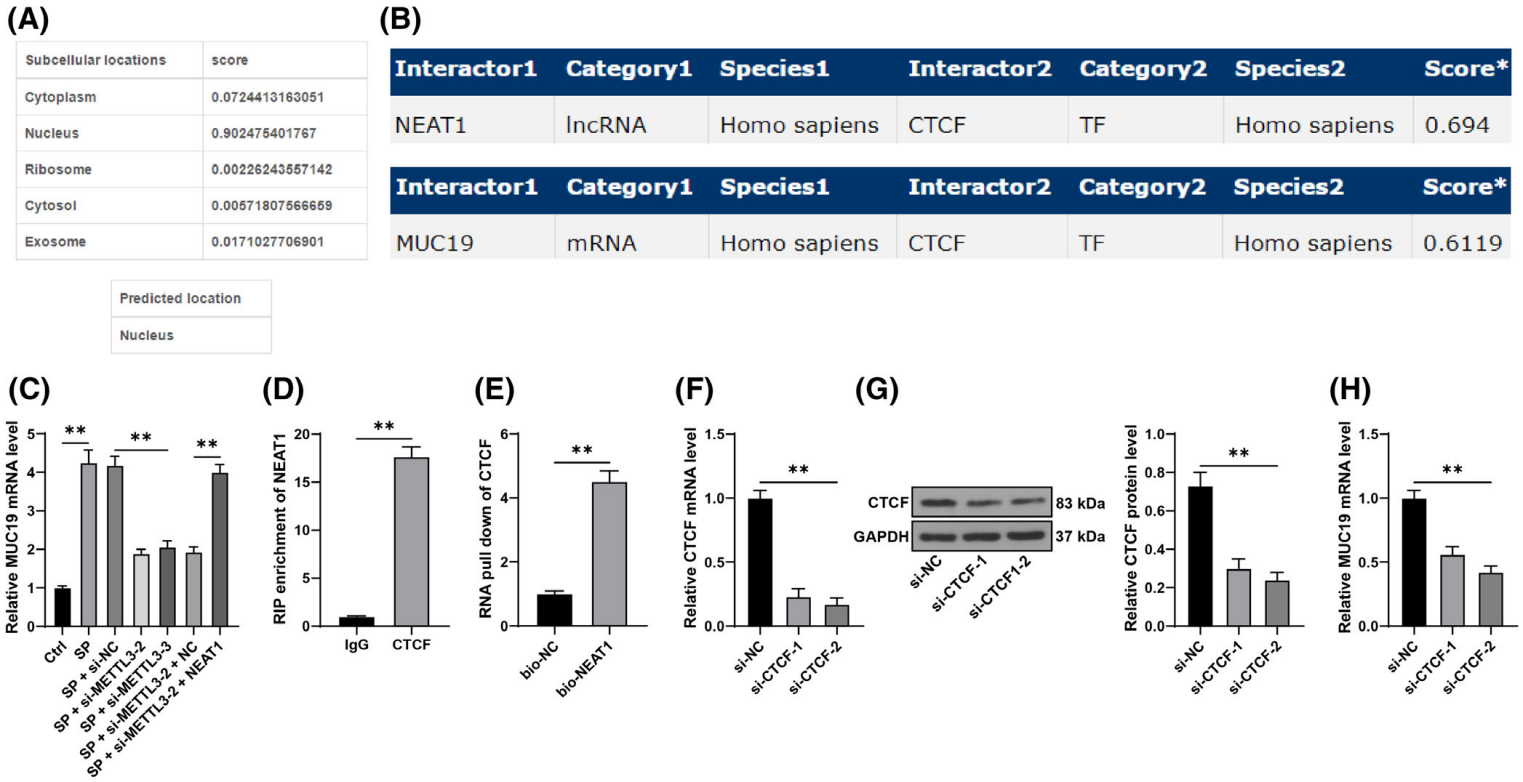
LncRNA NEAT1 binds CTCF and promotes the transcription of MUC19. (A): lncLocator database was used to predict the subcellular localization of NEAT1. (B): The RNAInter database was utilized to predict the binding relationships among NEAT1, CTCF, and MUC19. (C): MUC19 mRNA levels were measured by RT‐qPCR. (D, E): RIP and RNA pull‐down assays were performed to analyze the binding relationship between NEAT1 and CTCF. Cells were transfected with si‐CTCF while for the negative control, cells were transfected with si‐NC. (F, G): The intervention efficiency of CTCF was validated by RT‐qPCR and Western blot assay. (H): MUC19 mRNA levels were detected by RT‐qPCR. Each experiment was repeated thrice and the data were presented as mean ± standard deviation. Data comparisons between two groups in panels D and E were analyzed using the *t*‐test, while multiple group comparisons in panels C, F‐H were analyzed using one‐way ANOVA, followed by Tukey's multiple comparisons test. ** *p* < 0.01.

Prediction of NEAT1 localization was conducted using the lncLocator database (http://www.csbio.sjtu.edu.cn/bioinf/lncLocator/?tdsourcetag=s_pcqq_aiomsg), which revealed that NEAT1 is primarily located in the cell nucleus (Figure 5A). Furthermore, it has been revealed that NEAT1 binds to transcription factors and promotes the transcription of downstream genes.^14^ RNAInter database (http://www.rnainter.org/) predicted that NEAT1 can bind to CTCF, which can bind to MUC19 (Figure 5B). Previously, it has been observed that MUC19 downregulation alleviates lung viral infection diseases.^18^ Here, it was indicated that MUC19 transcription levels increased after SP induction and decreased after METTL3 silencing but increased again after NEAT1 overexpression (*p* < 0.05, Figure 5C). Based on this, it was hypothesized that the binding of NEAT1 to CTCF promotes the transcription of MUC19. RIP experiments showed significant enrichment in the CTCF immunoprecipitation group compared to the IgG immunoprecipitation group (*p* < 0.05, Figure 5D). Moreover, the RNA pull‐down experiments indicated stronger enrichment in the bio‐NEAT1 group compared to the bio‐NC group (*p* < 0.05, Figure 5E). To validate the role of CTCF in NEAT1‐mediated MUC19 upregulation, CTCF was knocked down in cells (*p* < 0.01, Figure 5F,G), which reduced the MUC19 transcription level (*p* < 0.01, Figure 5H). In conclusion, NEAT1 binds to CTCF and promotes MUC19 transcription.

### 3.6 MUC19 overexpression attenuates the protective effect of METTL3 downregulation on cell damage

**FIGURE 6:**
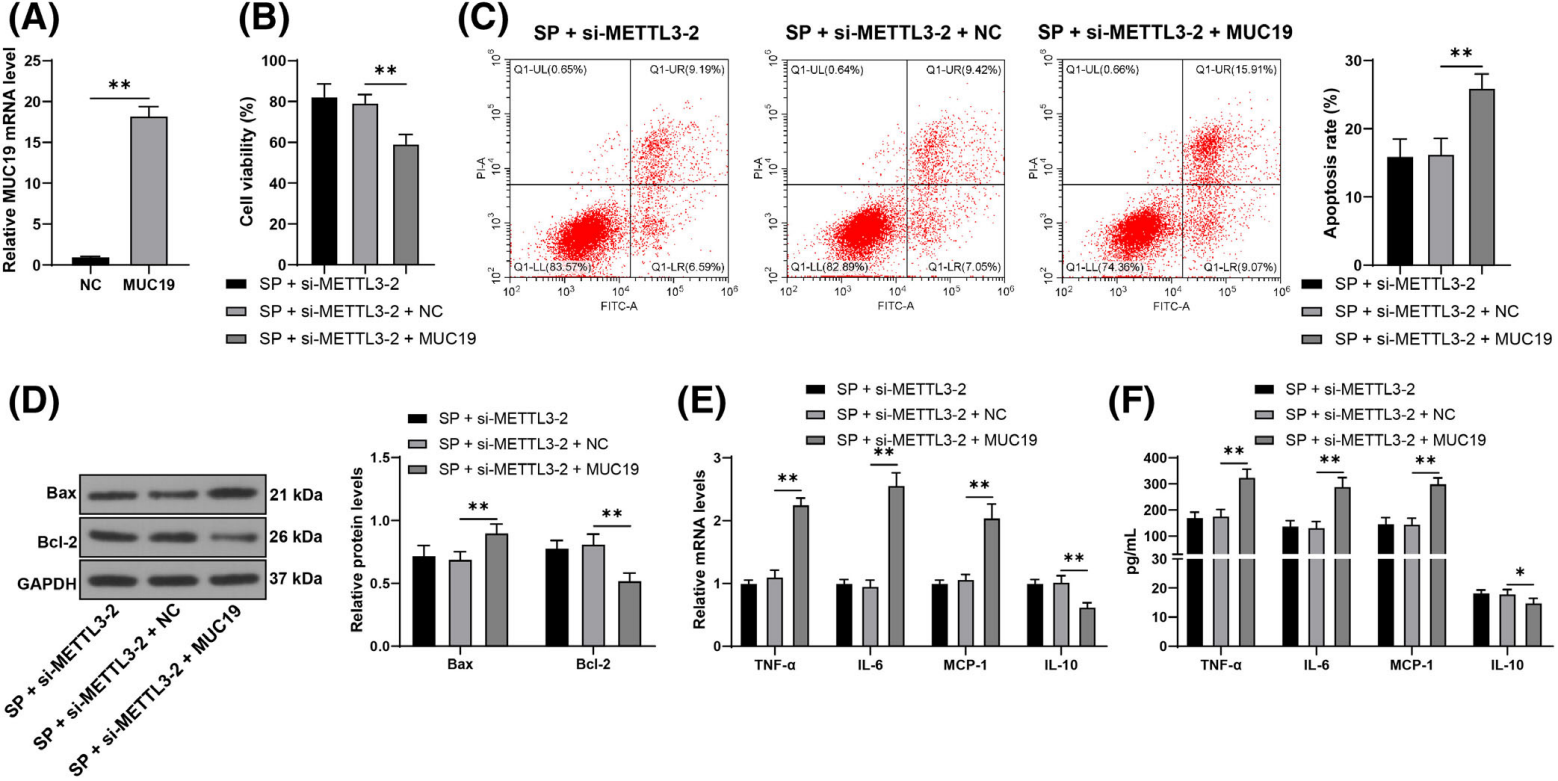
MUC19 overexpression attenuates the protective effect of METTL3 downregulation on cell damage. Cells were transfected with pcDNA‐MUC19 (MUC19), and the negative control was transfected with NC. AECs were infected with SP. (A): MUC19 mRNA levels were measured by RT‐qPCR. (B): Cell viability was assessed using CCK‐8 assay. (C): The apoptosis rate was determined by flow cytometry. (D): Levels of Bax and Bcl‐2 in cells were analyzed by Western blot assay. (E, F): Levels of TNF‐α, IL‐6, MCP‐1, and IL‐10 were measured by RT‐qPCR and ELISA. Each experiment was repeated thrice, and the data were presented as mean ± standard deviation. Data comparisons between the two groups in panel A were analyzed using a *t*‐test. Data comparisons among multiple groups in panels B and C were analyzed using one‐way ANOVA, while comparisons in panels D‐F were analyzed using two‐way ANOVA, followed by Tukey's multiple comparisons test. * *p* < 0.05, ** *p* < 0.01.

The above mechanism was validated by transfecting pcDNA‐MUC19 (MUC19) to upregulate the intracellular MUC19 transcription levels (*p* < 0.05, Figure 6A) and conducting combined experiments with si‐METTL3‐2. The results revealed that after upregulating MUC19 transcription, cell viability was decreased, while the apoptosis rate increased in the SP‐induced cells (*p* < 0.05, Figure 6B–D). Compared to the sole downregulation of METTL3, the combined experimental group showed increased levels of TNF‐α, IL‐6, and MCP‐1, and decreased levels of IL‐10 in cells (*p* < 0.05, Figure 6E,F). Overall, the data indicate that MUC19 overexpression attenuates the protective effect of METTL3 downregulation on cell damage induced by SP.

## 4 DISCUSSION

**FIGURE 7:**
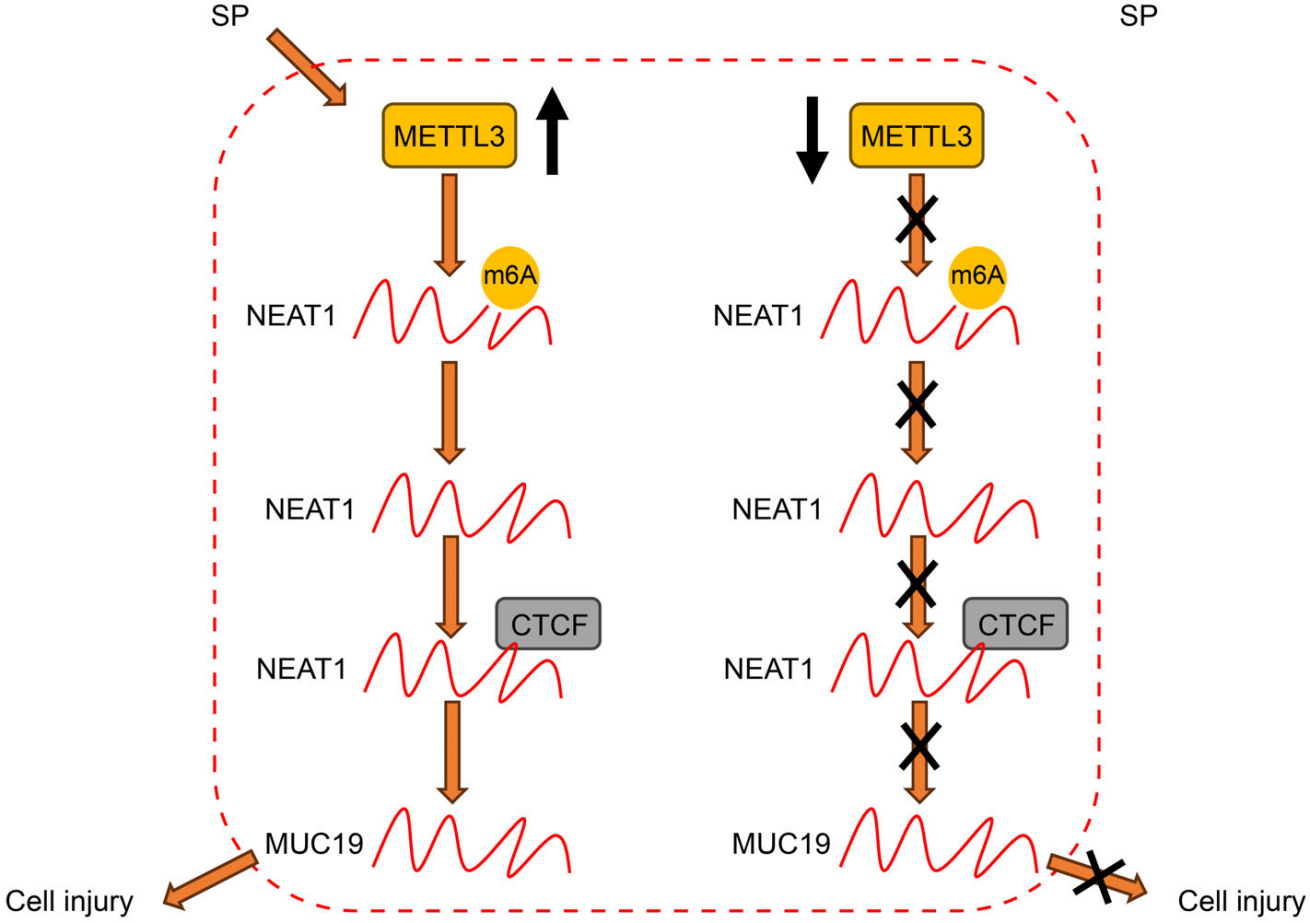
The mechanism of METTL3 in SP‐induced AEC injury in pneumonia. SP infection increases the expression of METTL3. METTL3 mediates m6A modification to increase NEAT1 expression and enhance its binding with CTCF, thereby promoting MUC19 transcription and ultimately causing apoptosis and inflammatory damage in AECs.

In ALI, m6A modification is closely associated with AEC injury, primarily regulating inflammatory cytokine release, cellular autophagy, and other morphological damages (alveolar septum thickening) in the lung.^20^, ^21^ As a methyltransferase, METTL3‐mediated m6A modification is widely involved in lung injury, inflammation, and pulmonary fibrosis.^9^, ^22^, ^23^ In this study, an SP‐induced pneumonia cell model was established, which revealed that METTL3 was highly expressed in AECs during pneumonia. METTL3‐mediated m6A modification could increase the expression of NEAT1, promote its binding with CTCF, facilitate MUC19 transcription, and ultimately trigger apoptosis and inflammatory damage in AECs (Figure 7).

In sepsis‐associated ALI, neutrophil extracellular trap production activates the ferroptosis signal, and the expression of METTL3 increases significantly to induce m6A modification of glutathione peroxidase 4, thereby causing ferroptosis in AECs and worsening lung injury.^23^ METTL3 gene knockdown can restore neprilysin expression and play an antiapoptosis and ALI‐protective role in AECs.^24^ Furthermore, it has been observed that METTL3 downregulation can reduce m6A modification on the enhancer of zeste homolog 2 (EZH2) mRNA, thereby decreasing EZH2 expression and inhibiting inflammation and cell apoptosis in a pneumonia cell model.^9^ Consistently, this study further validated that SP induced METTL3 upregulation, decreased AEC survival rate, increased apoptosis rate, reduced anti‐apoptotic factor (Bcl‐2), and elevated the levels of apoptotic factor (Bax) as well as inflammatory factors (TNF‐α/IL‐6/MCP‐1). Moreover, the abovementioned damage was alleviated after METTL3 downregulation. Therefore, METTL3 downregulation reduces SP‐induced pneumonia by alleviating apoptosis and inflammatory injury of AECs.

As a m6A methyltransferase, METTL3 essentially regulates different target genes via m6A methylation; therefore, these downstream genes should be elucidated to unveil the comprehensive molecular landscape of bacterial pneumonia. Extensive research indicates that NEAT1 potentially promotes the secretion of proinflammatory factors by activating microglial cells and has a regulatory role in the NF‐κB, AKT, TLR4, and TNF receptor‐associated factor 6 pathways.^11^ Moreover, NEAT1 was upregulated during ALI, and its knockdown attenuated LPS‐induced ALI by targeting the miR‐98‐5p/toll‐like receptor 4 (TLR4) axis.^25^ NEAT1 knockdown attenuated apoptosis of LPS‐induced AECs and also reduced the activities of LPS‐induced caspase‐3 and caspase‐9, pro‐inflammatory cell levels, and oxidative stress.^26^ A study indicated that induction of pneumonia in human embryonic lung fibroblasts upregulated NEAT1 expression, while transfection with si‐NEAT1 could increase miR‐146b expression, thereby enhancing cell viability and reducing apoptosis.^27^ Here, m6A quantitative analysis indicated that METTL3 silencing reduced the m6A level of NEAT1, suggesting that reduced m6A modification consequently inhibited NEAT1 expression. In addition, a series of experiments revealed that NEAT1 overexpression decreased cell viability and increased apoptosis in SP‐induced AECs, counteracting the protective effect of METTL3 silencing on damaged cells.

Mucins and mucus are essentially involved in the management of pulmonary diseases as they maintain lung homeostasis and protect against environmental threats using innate immune mediators.^28^ Herein, the RNAInter database predicted that NEAT1 can interact with the transcription factor CTCF, which can bind to MUC19. Further experiments revealed that NEAT1 binding with CTCF promoted MUC19 transcription. It has been suggested that in human alveolar basal epithelial cells, HMPV can induce MUC19 production,^29^ which is also involved in viral replication in the lungs of HMPV‐infected mice.^18^ Here, it was revealed that the SP induction elevated the MUC19 transcription levels, and MUC19 overexpression through intracellular transfection increased apoptosis and inflammatory factors in SP‐induced AECs. Another study reported that overexpression of MUC19 can reduce oxidative stress and inflammation levels in AECs by inhibiting the TLR4/NF‐κB signaling pathway.^30^ Therefore, it was hypothesized that MUC19 may play a role in SP‐induced AECs by regulating the TLR4/NF‐κB signaling pathway, which needs further validation.

### 4.1 Limitations

There are certain limitations in this study. (1) Only cellular‐level mechanisms were validated, and further validation through animal experiments and application in clinical research is required. (2) The changes in protein levels of MUC19 and its regulatory mechanisms were not assessed. (3) Many other molecular downstream mechanisms of METTL3 and the competing endogenous RNA mechanism of NEAT1 have not been fully investigated, requiring more experiments for exploration and validation. (4) Considering the current research funding and time constraints, the experiments on pneumonia induced by other bacteria were not included here. In the future, we plan to conduct animal experiments to further validate the mechanism, explore the downstream mechanisms of NEAT1, and continue the research on METTL3 and its mechanisms in pneumonia induced by other bacterial infections, to provide new knowledge for the treatment of bacterial pneumonia.

## 5 CONCLUSION

In summary, this study revealed that METTL3‐mediated m6A modification may increase NEAT1 expression, facilitate its binding with CTCF, and promote the MUC19 transcription, ultimately leading to apoptosis and inflammatory damage in AECs during lung injury. Furthermore, the revealed mechanism of the METTL3/NEAT1/CTCF/MUC19 axis provides new theoretical knowledge for bacterial pneumonia treatment and furnishes evidence for future therapeutic direction.

## CONFLICT OF INTEREST STATEMENT

The authors declare no conflict of interest.

## Supporting information


**Data S1.** Supporting information.
