# Supplementary material for: METTL3 aggravates cell damage induced by Streptococcus pneumoniae via the NEAT1/CTCF/MUC19 axis
Source: Kaohsiung J Med Sci. 2024 May 16;40(8):722–31. doi: 10.1002/kjm2.12843 (PMC11895658; doi:10.1002/kjm2.12843)
Supplement: Supplementary file 1 — Data S1. Supporting information. [file KJM2-40-722-s001.pdf]

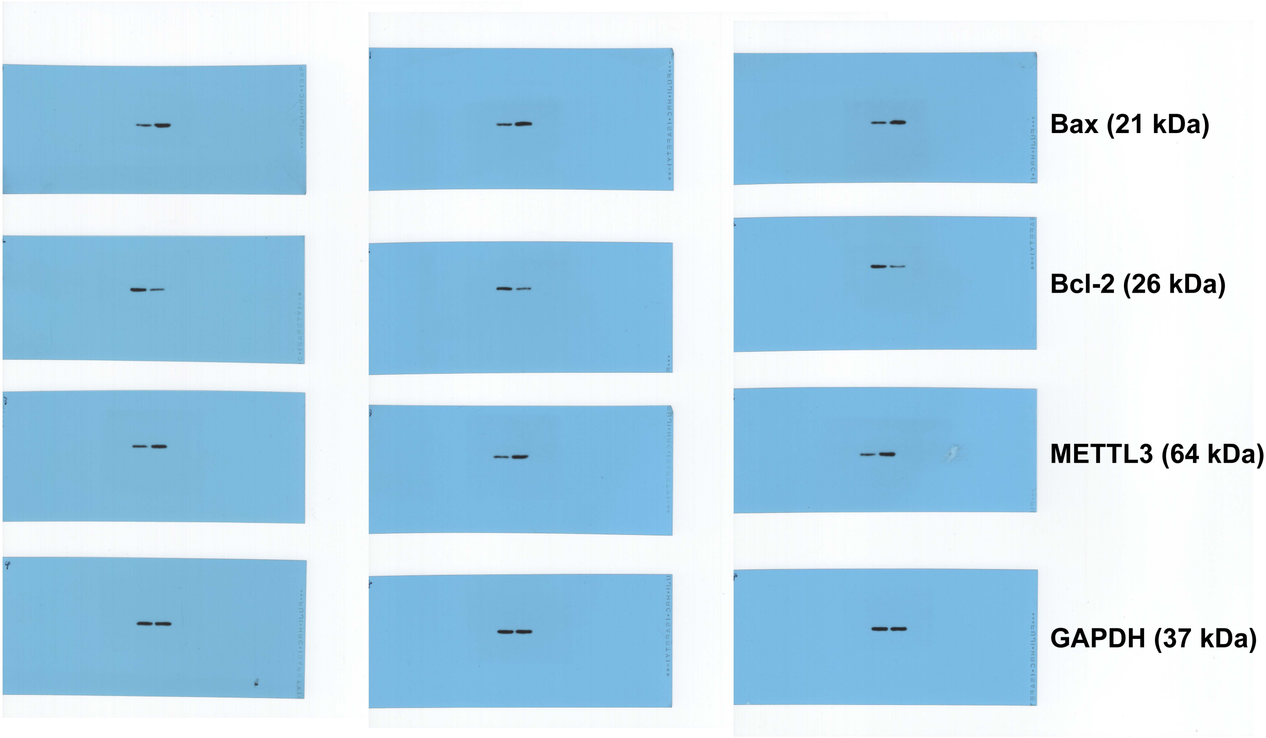
Bax (21 kDa)
Bcl-2 (26 kDa)
METTL3 (64 kDa)
GAPDH (37 kDa)

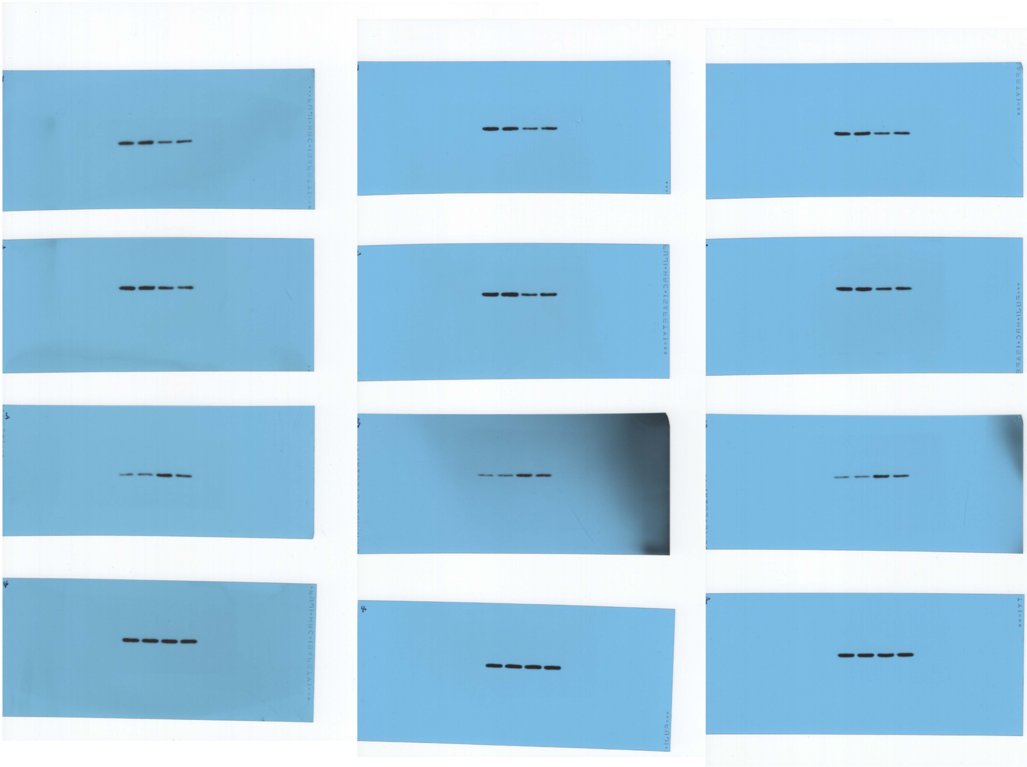

METTL3 (64 kDa)

Bax (21 kDa)

Bcl-2 (26 kDa)

GAPDH (37 kDa)

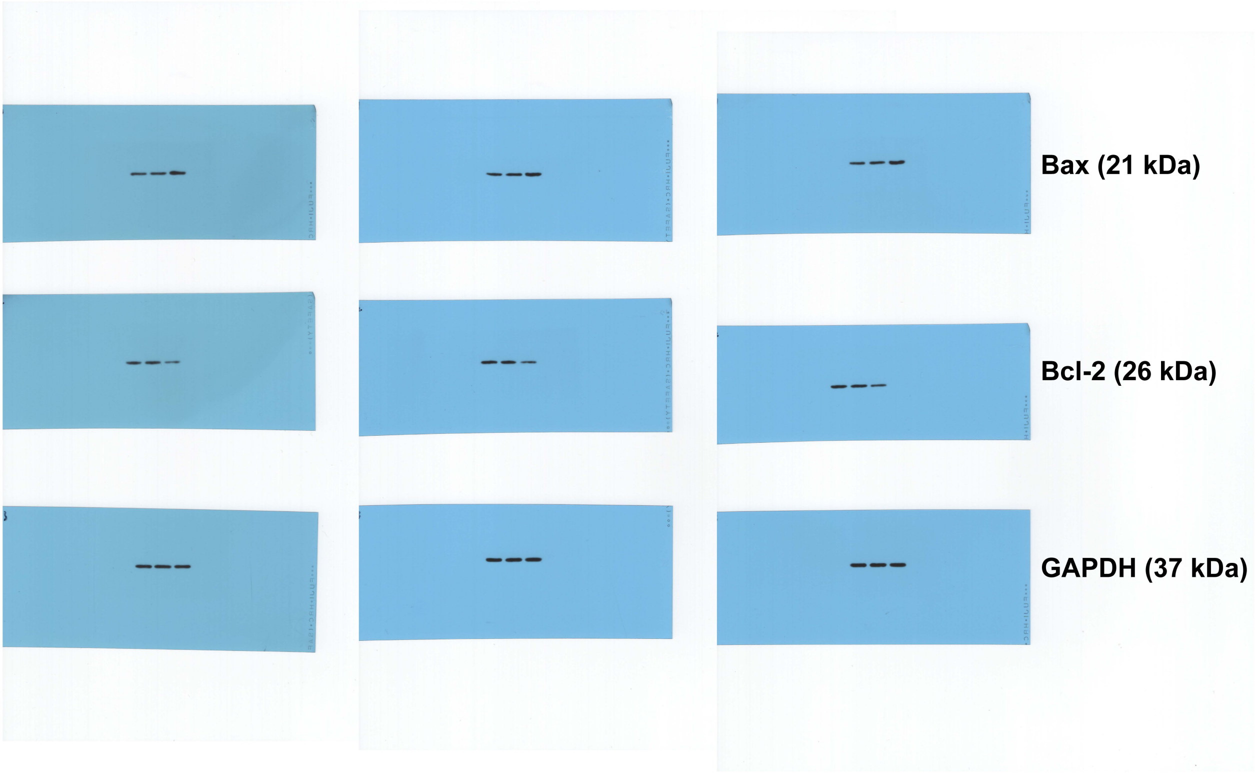
Bax (21 kDa)
Bcl-2 (26 kDa)
GAPDH (37 kDa)

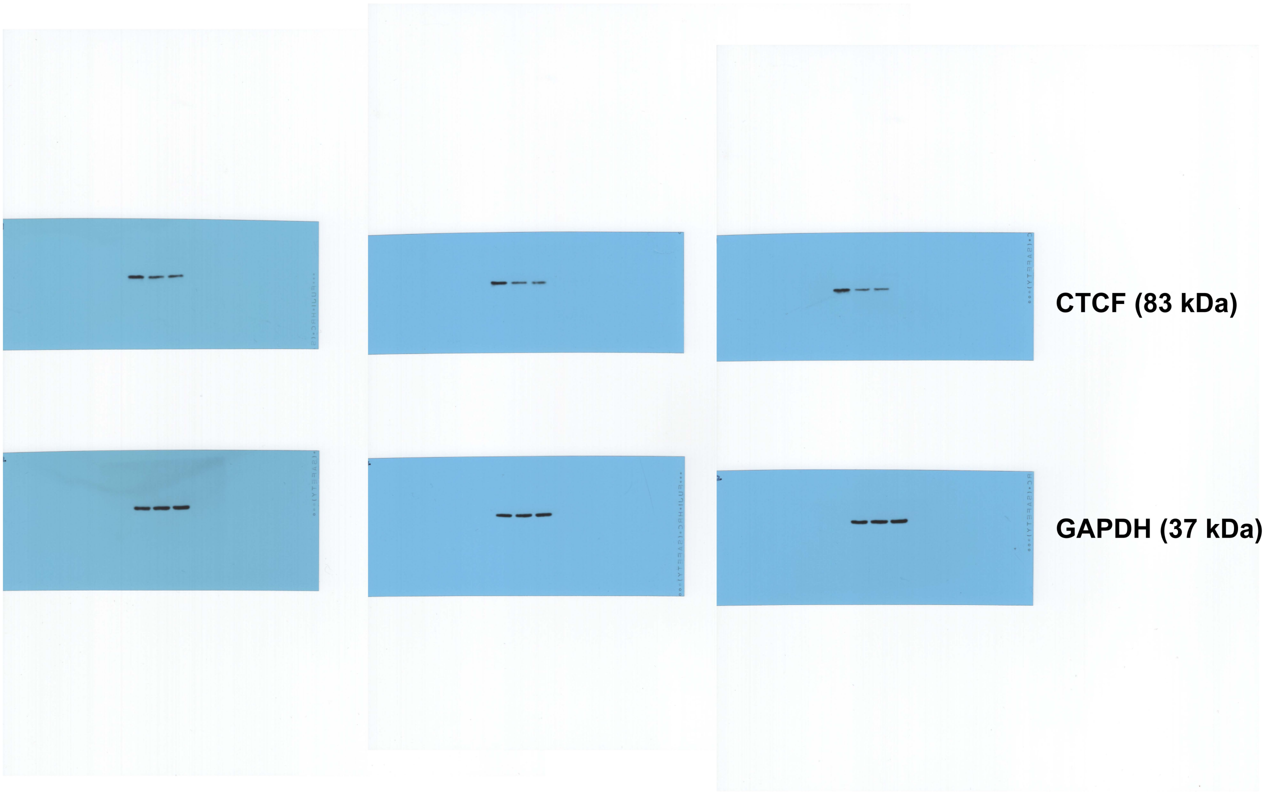
CTCF (83 kDa)
GAPDH (37 kDa)

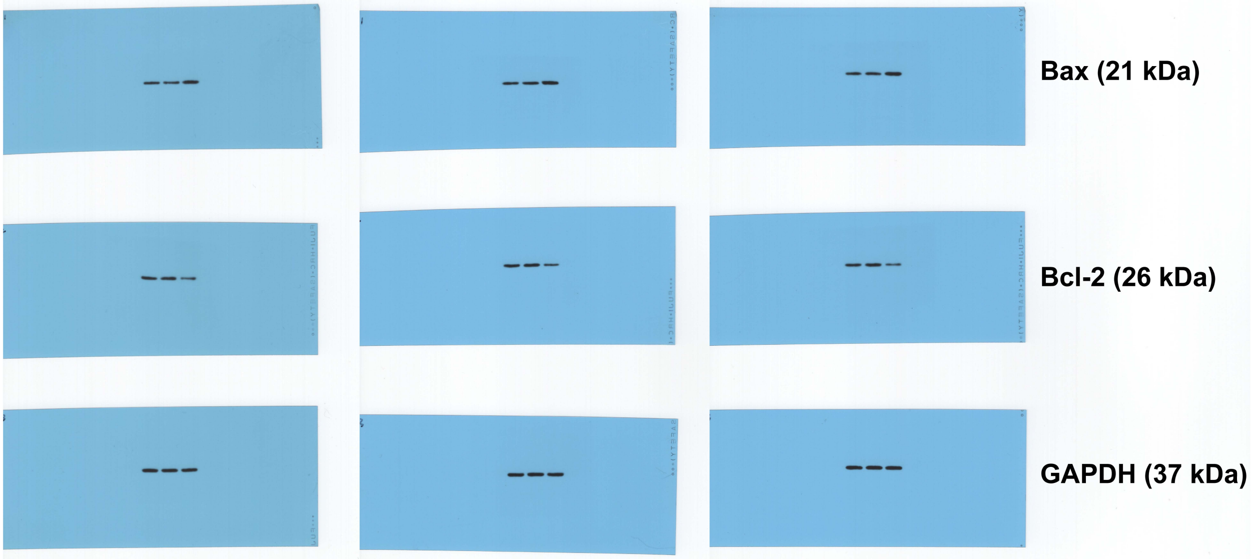
Bax (21 kDa)
Bcl-2 (26 kDa)
GAPDH (37 kDa)
